# Complete Rupture of the Perineum, Etc.

**Published:** 1890-12

**Authors:** W. R. Blailock

**Affiliations:** M’Gregor


For Daniel's Texas Medical Journal.

### COMPLETE RUPTURE OF PERINEUM—TREATMENT.

BY W. R. BLAILOCK, M. D., M'GREGOR.

OCTOBER 18th, 10 p. m., I was called to a perimpara. During the latter part of gestation diarrhœa had been very troublesome. Pains came on during forenoon of 18th and were increasing perceptibly in frequency and severity. Examination revealed os dilated to size of a silver dollar, vertex presentation, occiput to left acetabulum.

October 19th, 2 o'clock a. m., os well dilated, membranes ruptured with finger nail.

Pains grew stronger and it seemed that two hours at most would be sufficient for the termination of labor. The head descended into lower strait and remained without any change until 10 o'clock a. m. At this time the fœtal heart had become very feeble and the mother was showing signs of exhaustion. Applied forceps and made traction during pains, guarding the perineum as much as possible. In about twenty minutes a pain of more power came on, or else more traction than was prudent was made and the child's head and shoulders came out before the forceps could be unclasped. A little resuscitating brought the child round all right, which was a ten pound girl.

The placenta was delivered with comparative ease, but in spite of a teaspoonful of fluid ext. ergot given as soon as placenta was delivered, she had a severe hemorrhage, which was controlled by compression.

On examination, as might have been expected, a complete rupture of perineum was found. The sphincter muscle being torn through, the rent extending 2½ inches up the rectum. The patient was now rallying from the throes of labor and loss of blood, and I determined to restore the gut and perineum at once, if possible. The vagina was irrigated with water as warm as could be borne; the buttocks and rectum cleaned with warm water. The gut was stitched from above downward to sphincter, interrupted cat gut sutures being used. The two ends of sphincter were neatly approximated by means of stouter cat gut sutures passed from below, one near the upper, the other near the lower border of that muscle, leaving the knots on rectal side. Two deep perineal sutures of silk completed the operation.

A large drainage tube, well penetrated in its upper third, was passed well up into posterior cul-de-sac, iodoform gauze packed well up against the parts covering vulva and rectum; over this a thick layer of absorbent cotton, not including end of drainage tube, and the whole secured by bandage. The end of drainage tube bent downward was covered by additional gauze and cotton so the lochial discharge could be removed twice daily.

The urine was drawn with soft catheter once a day, until fourth day she passed water before my arrival. The dressing was now removed and found perfectly free from putrefactive odor. A fresh dressing was applied, the same as the first, and removed every day, the drainage tube was removed on the fifth day and left out.

Bowels were moved on the fifth day by introducing a flexible catheter attached to fountain syringe, up above sigmoid flexure of colon, and letting about a quart of warm water run in.

The first action revealed the existence of a small recto-vaginal opening. Vaginal douches of warm water were used once a day from this time, and by the twelfth day the recto-vaginal opening was entirely closed. Perineal sutures were removed on the twelfth day. Union of gut, sphihcter and perineum perfect.

Patient had a slight rise of temperature on the third day, which I attributed to the establishment of lactation, as there was neither tenderness nor swelling of uterus, ovaries nor bowels.

Nothing new is claimed for this case. I have simply given the different steps in the treatment where immediate operation was followed by a most happy result. Involution has progressed nicely, and the lady at this time, thirty days after operation, is up and doing well, and delighted with her thriving stout girl baby.

I have used forceps a score or more times, and this is my first complete laceration. I possibly could have avoided this, but then the child would doubtless have died.

